# Use of bronchoalveolar lavage in diagnosing angioimmunoblastic T‐cell lymphoma: A case report

**DOI:** 10.1002/rcr2.924

**Published:** 2022-03-03

**Authors:** Gaku Yamamoto, Kei Takamura, Yuriko Ishida, Yuma Sato, Ayuka Sinozaki, Hajime Kikuchi, Makoto Yamamoto, Hajime Kobayashi, Naoki Hirose, Keisuke Kikuchi

**Affiliations:** ^1^ Department of Respiratory Medicine Obihiro Kosei Hospital Obihiro Japan

**Keywords:** angioimmunoblastic T‐cell lymphoma, bronchoalveolar lavage

## Abstract

Angioimmunoblastic T‐cell lymphoma (AITL) is a type of peripheral T‐cell tumour that belongs to the group of non‐Hodgkin's lymphomas. Pulmonary lesions can be found in 7%–10% of AITL cases. Imaging findings of the lungs varied; however, immunoblastic infiltration in the lungs is rare. Our patient was a 73‐year‐old man who received repeated chemotherapy for AITL. Fourth‐line therapy using romidepsin controlled the illness, but the patient was hospitalized for dyspnoea and an infiltrative shadow. We performed bronchoalveolar lavage (BAL), and the culture was positive for *Haemophilus influenzae*. The patient was initially discharged with antibiotic therapy, but hospitalized again. Antibiotics were ineffective and the patient required mechanical ventilation. BAL was performed again, after which fluid cytology revealed immunoblast‐like atypical cells. Therefore, the patient was diagnosed with pulmonary infiltration due to AITL. Steroid therapy proved ineffective, and the patient died. BAL was used to effectively diagnose pulmonary AITL infiltration.

## Graphical abstract

To our knowledge, we report here the first case of angioimmunoblastic T‐cell lymphoma in which pulmonary involvement was detected using bronchoalveolar lavage.

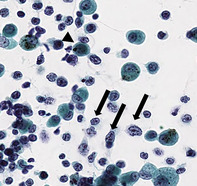

## INTRODUCTION

Angioimmunoblastic T‐cell lymphoma (AITL) is a peripheral T‐cell neoplasm classified as a moderate grade non‐Hodgkin's lymphoma.^1^ AITL is a rare disease, occurring in 2%–4% of non‐Hodgkin's lymphomas, and causes pulmonary disease in approximately 7%–10% of cases.^2^ Lymphocytes and plasma cell infiltration are usually detected on histological examinations; however, immunoblastic cell invasion is rare. To our knowledge, pulmonary lesions in which tumour cell invasion is medically confirmed during the patient's lifetime have been reported in only six cases. Here, we report the first case of AITL to our knowledge in which pulmonary involvement was detected using bronchoalveolar lavage (BAL).

## CASE REPORT

**FIGURE 1:**
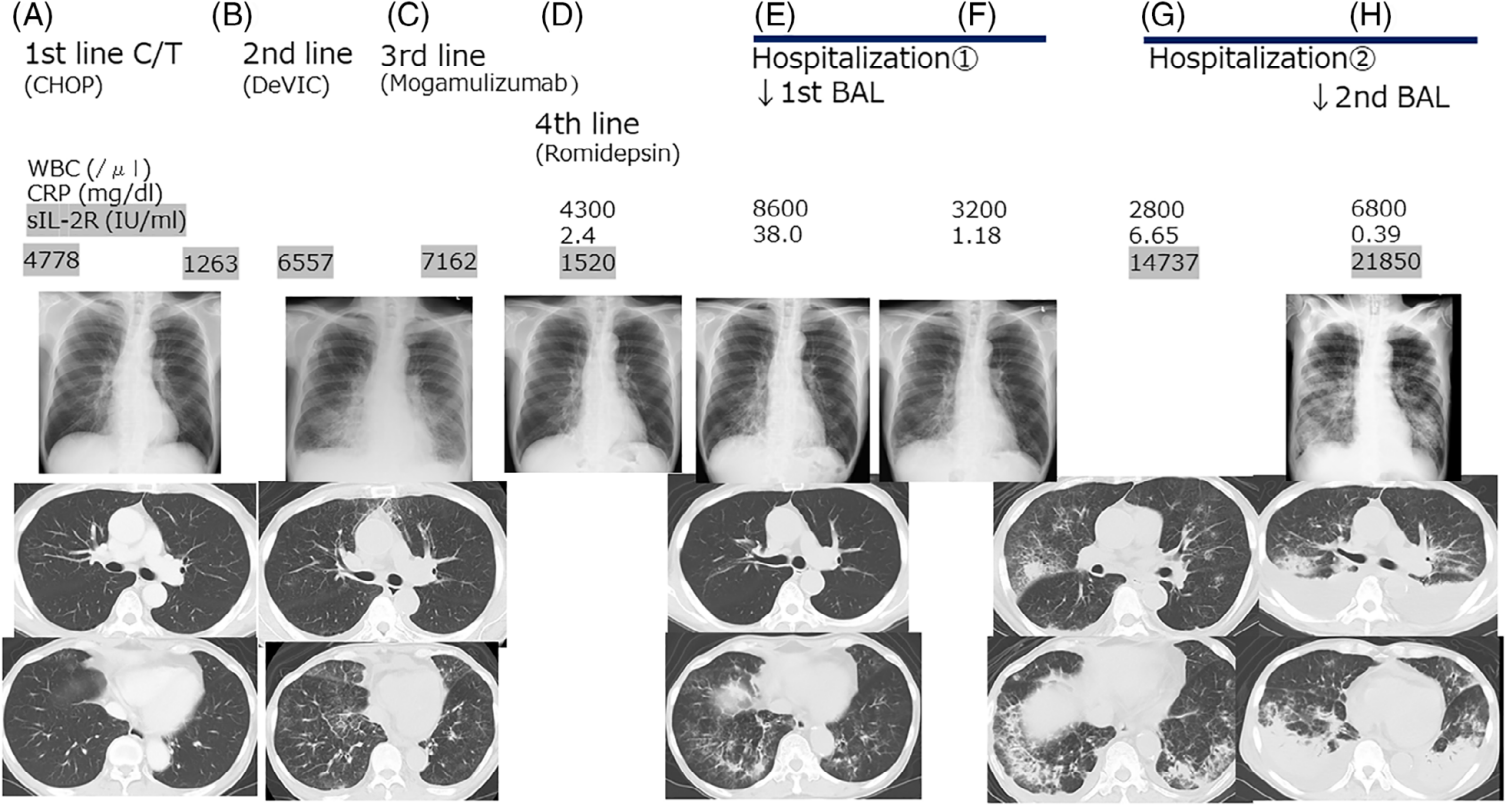
Clinical course. (A) CHOP therapy was started as the first‐line chemotherapy. (B) Second‐line chemotherapy (DeVIC) was ineffective. (C) Third‐line chemotherapy (mogamulizumab) developed drug‐related interstitial lung disease. (D) Fourth‐line chemotherapy (romidepsin) was effective, and was administered for several courses. (E) An infiltrative shadow was found in both lower lobes of the lung and first bronchoalveolar lavage (BAL) was performed. (F) The patient was diagnosed with bacterial infection caused by *Haemophilus influenzae* and discharged after ceftriaxone administration. (G) The patient was re‐admitted for dyspnoea and bilateral infiltrative shadow in the chest x‐ray on the day before the planned romidepsin therapy was due. (H) We performed BAL again and immunoblast‐like atypical cells were found in the cytology

A 73‐year‐old man was diagnosed with AITL via biopsy of the left inguinal lymph nodes, and CHOP therapy (cyclophosphamide, doxorubicin, vincristine, prednisolone) was started as first‐line chemotherapy (Figure 1A). The patient responded well to the treatment. However, increased serum soluble interleukin‐2 receptor levels (sIL‐2R, 6557, reference range: 122–496 U/ml) and re‐swelling of both lymph nodes of the groin were observed. Second‐line chemotherapy (DeVIC; dexamethasone, etoposide, ifosfamide, CBDCA) was ineffective (Figure 1B), and we initiated mogamulizumab as the third‐line chemotherapy; however, the patient developed drug‐related interstitial lung disease (Figure 1C), and chemotherapy was discontinued while prednisolone was started. After prednisolone was reduced to a maintenance dose, we started romidepsin as the fourth‐line chemotherapy (Figure 1D). Romidepsin was effective, and was administered for several courses, but the patient was hospitalized due to dyspnoea (Hospitalization 1). An infiltrative shadow was found in both lower lobes of the lung on chest computed tomography (CT) (Figure 1E); thus, we performed BAL from the right B8b, which showed elevated levels of neutrophilic fractionation, and the culture was positive for *Haemophilus influenzae* (Table 1A). *Haemophilus influenzae* was also detected in the blood and sputum. Thus, we diagnosed the patient with a bacterial infection caused by *H*. *influenzae*. Ceftriaxone was administered, and the patient was discharged (Figure 1F).

**TABLE 1:** Clinical course of BAL

	(A)	(B)
	First BAL	Second BAL
Total cell (/μl)	1655	230
Neutrophil (%)	92	24
Eosinophil (%)	0	0
Lymphocyte (%)	6	54
Macrophage (%)	2	22
Cytology	Class I	Class V
Bacteria	*Haemophilus influenza*	*Streptococcus* sp.
Site	rt.B8b	rt.B2a
Recovery (ml)	20/100	90/150

Abbreviations: BAL, bronchoalveolar lavage; rt, right.

**FIGURE 2:**
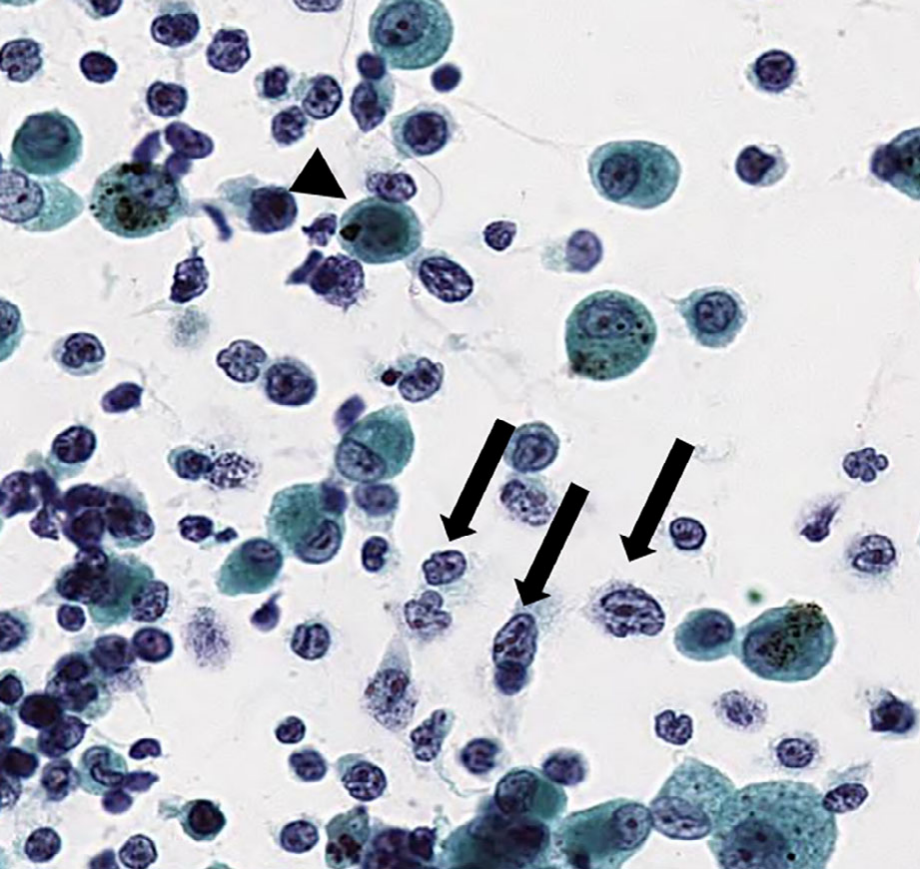
Cytology findings of bronchoalveolar lavage fluid. Colonization of atypical lymphocytes with enlarged nucleolus and immunoblastic‐like atypical cells (arrows) were detected, and also atypical plasma cells (arrowhead) were found

We planned therapy with romidepsin again; however, the patient was re‐admitted for dyspnoea and bilateral infiltrative shadow in the chest x‐ray on the day before romidepsin therapy was due (Hospitalization 2). Laboratory examinations at second admission showed elevated levels of C‐reactive protein (6.65 mg/dl, reference range: <0.3 mg/dl) and sIL‐2R (14,737 IU/ml; Figure 1G). White blood cell counts were slight decreased (2800/μl, reference range: 3600–9000/μl). Infiltrative shadows appeared in both upper lobes, and exacerbation of the infiltrative shadow in both lower lobes of the lung was observed (Figure 1G). We considered bacterial pneumonia again, and cefozopran was started; however, the patient was intubated and mechanical ventilation was performed on the eighth day of admission. Exacerbation of the infiltrative shadow in both upper lobes of the lung and significant bilateral pleural effusion were detected (Figure 1H). We performed BAL again to differentiate the progression of AITL from concurrence of the infection. BAL from the right B2a showed elevated levels of lymphocytic and neutrophilic fractionation; however, bacteria were not detected (Table 1B). Colonization of medium to large atypical lymphocytes with enlarged nucleoli, immunoblast‐like atypical cells and plasma cells of atypical cells were found in the cytology results (Figure 2). Evidence of malignant lymphoma was obtained, and the patient was diagnosed with pulmonary infiltration of the AITL. Steroid pulse therapy was started the next day, but the effect was not observed as the patient died from the illness.

## DISCUSSION

AITL is a peripheral T‐cell lymphoma characterized by lymph node invasion by various lymphocytic infiltrations with hyperplastic endodermis veins and follicular dendritic cells.^1^

Sugiyama et al.^2^ reported that pulmonary involvement of AITL presents various findings; however, pulmonary involvement of AITL was diagnosed based on imaging findings.^2^ The cases in which histological evaluations were available only showed local infiltration of lymphocytes and plasmacytes.^2^ Almost all cases in which invasion of the immunoblastic cells was confirmed histologically were dependent on autopsy.^2^ Table 2 shows cases of AITL with pulmonary involvement in which immunoblastic cell invasion was confirmed during the patient's lifetime. We describe the imaging findings and diagnostic methods. A conventional report stated that imaging findings varied. Pulmonary involvement was observed in four previous cases.^3^, ^4^, ^5^ To our knowledge, this is the first case that led to a diagnosis of AITL with pulmonary infiltration using BAL. In the present case, we performed BAL twice (Table 2). To our knowledge, a diffuse increase in infiltrative shadow was observed on CT for the first time. BAL findings revealed an infectious status, and antibiotic therapy improved the patient's general condition. However, the clinical manifestations worsened again, and he was finally diagnosed with pulmonary infiltration due to AITL after BAL was performed again. The imaging findings showed rapid exacerbation of infiltrative shadows and onset of bilateral effusions, suggesting rapid AITL progression. A difference in disease activity may affect the detection of malignant cells.

**TABLE 2:** Diagnostic cases of AITL with pulmonary involvement during the lifetime

Case	Year	Author	Age	Sex	Radiology findings				Diagnosis	
					LA	PE	Int S	AlS	Method	Lesion
1	1976	Iseman et al.^3^	66	F	(−)	(+)	(+)	(−)	OLB	Lung
2	1976	Zylak et al.^4^	57	F	(+)	(−)	(+)	(+)	OLB	Lung
3	1976	Zylak et al.^4^	67	F	(+)	(−)	(+)	(+)	TBLB	Lung
4	1977	Asher et al.^5^	78	F	(+)	(+)	(+)	(+)	TBLB	Lung
5	2020	Our case	74	M	(−)	(+)	(+)	(+)	Cytology	BALF

Abbreviations: AITL, angioimmunoblastic T‐cell lymphoma; AlS, alveolar shadow; BALF, bronchoalveolar lavage fluid; F, female; Int S, interstitial shadow; LA, lymphadenopathy; M, male; OLB, open lung biopsy; PE, pleural effusion; TBLB, transbronchial lung biopsy.

## CONFLICT OF INTEREST

None declared.

## ETHICS STATEMENT

The study was approved by the Ethics Committee of Obihiro Kosei General Hospital 2020‐010.

The authors declare that appropriate written informed consent was obtained for publication of this manuscript and accompanying images.

## Data Availability

The data that support the findings of this study are available from the corresponding author upon reasonable request.
